# Reverse Peroneal Artery Flap for Large Heel and Sole Defects: A Reliable Coverage

**Published:** 2017-05

**Authors:** Yogesh C. Bhatt, Sumer Singh, Piyush Doshi, Sanjay G. Vaghani

**Affiliations:** Department of Plastic and Reconstructive Surgery, SumandeepVidyapeeth University, Vadodara, India

**Keywords:** Peroneal artery flap, Sural flap, Foot, Heel, Reconstruction

## Abstract

**BACKGROUND:**

Large soft tissue defects of ankle and foot always have been challenging to reconstruct. Reverse sural flaps, free flaps have been used for this problem with variable success. Reverse peroneal artery flap is an option to use with reliability without microvascular repair. Connections of peroneal artery around talus and ankle joint are deep and reliable with anterior tibial and posterior tibial artery. Arterial inflow and venous drainage improved with including short saphenous vein and reverse sural artery in the flap.

**METHODS:**

Ten patients with large defects around heel underwent reconstruction with (RPAF) reverse peroneal artery flap (pedicled) over a period of 2 years. Final inset given after 18-21 days of primary surgery. The mean age of these patients was 45 years.

**RESULTS:**

Of the 10 flaps, all showed complete survival without even marginal necrosis. Two patients had minor donor site problems that settled with conservative management.

**CONCLUSION:**

RPAF is a very reliable flap for the coverage of large soft tissue defects of the heel and sole. Large defects can be reconstructed without microvascular surgery and without compromising major vessel of foot region. If some experience with perforator flaps and free fibula is there then RPAF is easy to execute with reliability.

## INTRODUCTION

Complex soft tissue defects around the foot represent a difficult reconstructive problem due to exposure of the bones, joints and tendons. Multiple reconstructive techniques have been proposed to repair soft tissue defects in these regions including local cutaneous flaps, pedicled fascial or fasciocutaneous flaps, pedicled muscle flaps and free flaps.^1^^,^^2^ Free tissue transfers provide a large amount of soft tissue at the most desired places, and are quiet reliable in experienced hands. But, non-availability of microsurgical expertise and facility at peripheral centres, the cost and, sometimes, the patient-related factors may preclude the option of the microvascular surgery.^3^

Although free flaps plays an important role in limb salvage, better understanding and applications of regional flap designs have sometimes provides easier and more cost-effective alternatives for soft tissue coverage of the injured lower extremity.^4^ Local flaps in the foot have limitations of reach and reduced amount of soft tissue that can be used. Donski and Fogdestam^5^ described distally based fasciocutaneous flap based on the perforators of the peroneal artery around the ankle region and their communications with the superficial sural artery. 

This flap, with its modifications, has been used for small to medium sized defects.^6^ Multiple deep communications exist between the peroneal artery and the tibial arteries (both anterior and posterior) around ankle joint, as demonstrated by Cormack and Lamberty.^7^ Reverse peroneal artery flaps (RPAF) is based on these communications. The skin territory supplied by the distal constant peroneal perforator which is generally located 5-7 cm above the lateral malleolus, is limited. This limits its dimensions, pedicle length and the distal reach in reverse manner.^7^

To gain distal reach in reverse manner to heel and more distal sole we need to include peroneal artery in the flap axis. The peroneal artery is the blood supplying vessel to lateral and posterior skin of leg via perforators. Inclusion of the artery with its proximal perforators helps to increase flap length as we need. The problem of venous drainage is improved by fasciocutaneous pedicle and by inclusion of the short saphenous vein in the flap. The distance between peroneal artery and short saphenous vein in proximal leg is quite more but in large defects to reconstruct it is helpful as we can take more skin between lateral and posterior calf.^7^

Advantage of the reverse sural artery is also taken by including short saphenous and sural artery system in the flap. In this study, the reverse sural flap design was modified to include the peroneal artery with its perforator in the leg. This makes the flap completely robust and reliable, allowing inclusion of the entire posterior calf skin as well as increasing its reach. As there is a good fasciocutaneous pedicle, short saphenous vein and two big venae comitants, the venous congestion will never been a problem.^7^ The present paper gives detailed description of the reverse peroneal artery flap design, dissection of the flap and the results in our 10 cases.

## MATERIALS AND METHODS

A total of 10 patients with soft tissue defects over the heel and distal sole underwent reconstruction by RPAF transfer in our institute (Sumandeep Vidyapeeth University) between 2013 and 2015. Details like history, age, sex, etiology, dimensions of defect, reason for flap cover, complications and hospital stay were noted from patients’ medical records.Pre-operativelyany history of previous trauma/surgery to the limb, etiology, the site and the size of the defect and peripheral pulses were assessed. This flap was planned when at least one of the major vessels (ATA/PTA) is palpable at level of ankle.

There isno need to localize peroneal perforators preoperatively. All the procedures were undertaken under spinal anesthesia. All surgeries were done under tourniquet control after elevating lower limb for 4-5 minutes. Preparation of the defect along with elevation of the flap was done under single permissible tourniquet time (near about 1.5 hours). Initial 4 surgeries were done in supine position with knee flexed but later on we found that prone position is far better for defect preparation, flap elevation, flap insetting and donor site grafting. So the next 6 surgeries were done in prone position.

**Fig. 1:**
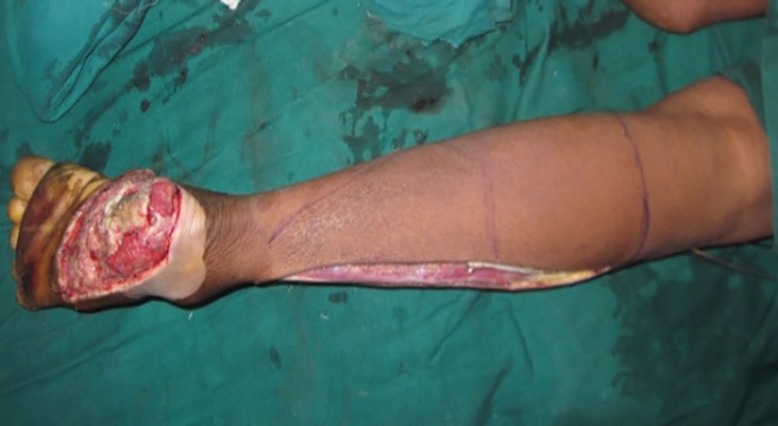
Defect preparation and flap marking of the reverse peroneal artery flap. Marking was planned in reverse. Base of the flap was between tendoachilles and lateral malleolus and 5 cm above the lateral malleolus. Lateral border of the flap marked over fibula and medial border marked in such a way that short saphenous system included in the flap. Fibular side of the flap dissected and peroneal vessels ligated at this stage after confirming the entrance of the proximal perforators in to the flap

The flap was planned in reverse with pivot point 5 cm above the lateral malleolus with a narrow base between lateral malleolus and tendoachilles. To avoid injury to the important communicating vessels, an area of 5 cm above the lateral malleolus is kept undisturbed. The flapcan be safely extended to the upper end of the leg, up to the line of the knee joint and laterally up to the mid lateral line on either side (Figure 1) according to need. Incision was taken straight down deep to the fascia on the marked lateral side(fibular side) of the flap. The perforators from peroneal artery were seen entering to skin at this stage. These are reasonably constant in the middle third of the leg. 

**Fig. 2:**
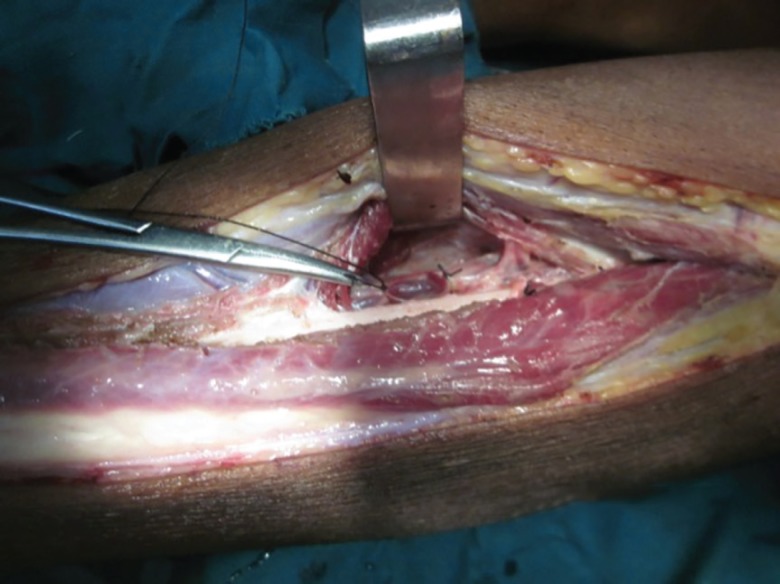
After elevating the fibular side of the flap, peroneal perforators identified and peroneal vessels ligated proximal to the most proximal perforator entering in to the flap. A big perforator seen entering in to the flap just below artery forceps

**Fig. 3:**
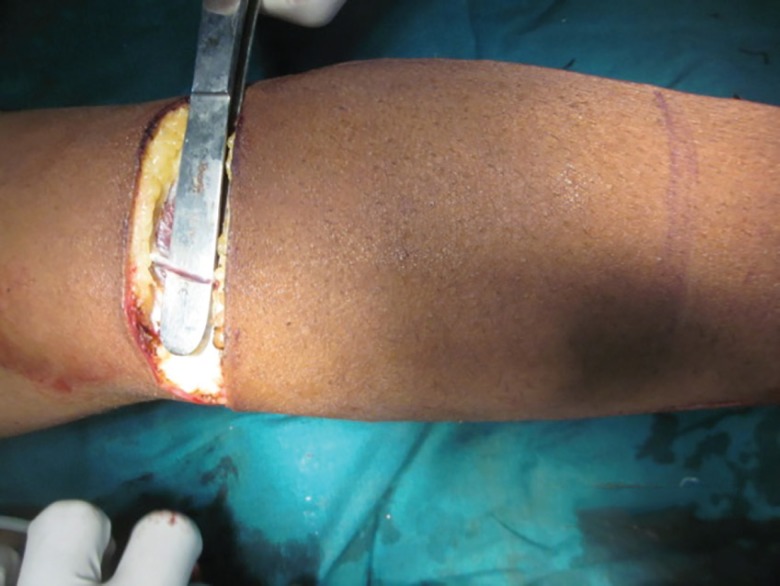
Sural vessels with the sural nerve and short saphenous vein taken in to the flap. Short saphenous vein is located in subcutaneous plane here but sural artery is located between muscle bellies of gastrocnemius below fascia

We select most proximal and big perforator to include in our flap. Then dissection is done deep to peroneus muscles separating them from flap and fibula. Periosteum of this side fibula separated from bone through which we directly go to the peroneal artery (Figure 2). Now upper end of the flap dissected subfascially and short saphenus vein along with sural artery and sural nerve were included in the flap (Figure 3). Medial side of flap then subfascially elevated till (septum) those perforators which were initially identified. Now peroneal artery with venae comitantes ligated proximally and then flap elevated from here to distal side (Figure 2). 

**Fig. 4:**
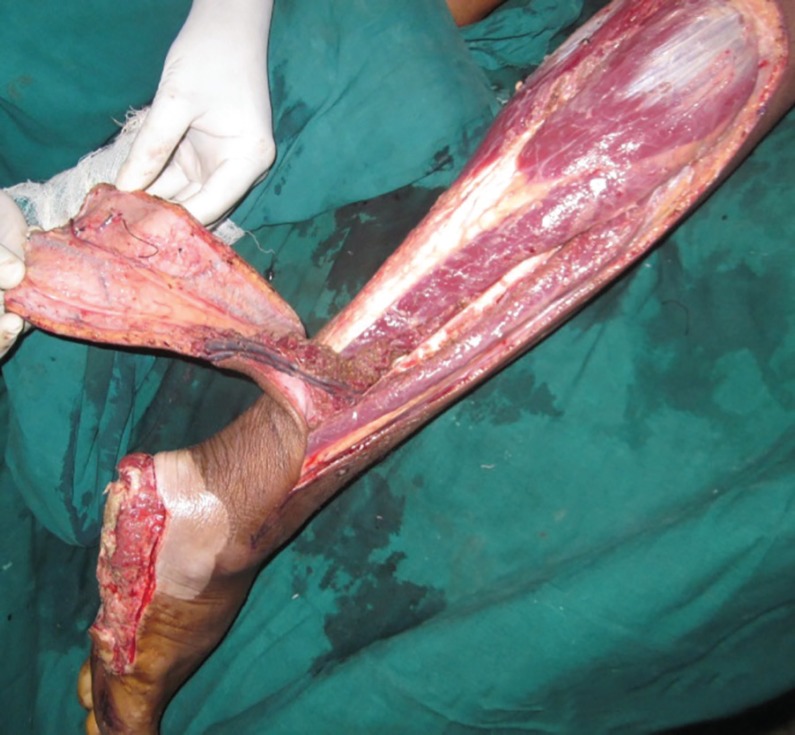
Elevated flap with the peroneal vessles and part of the flexor hallucis longus muscle. Sural vessels, short saphenous vein, peroneal vessels and tendoachilles with paratenon seen in the picture

**Fig. 5:**
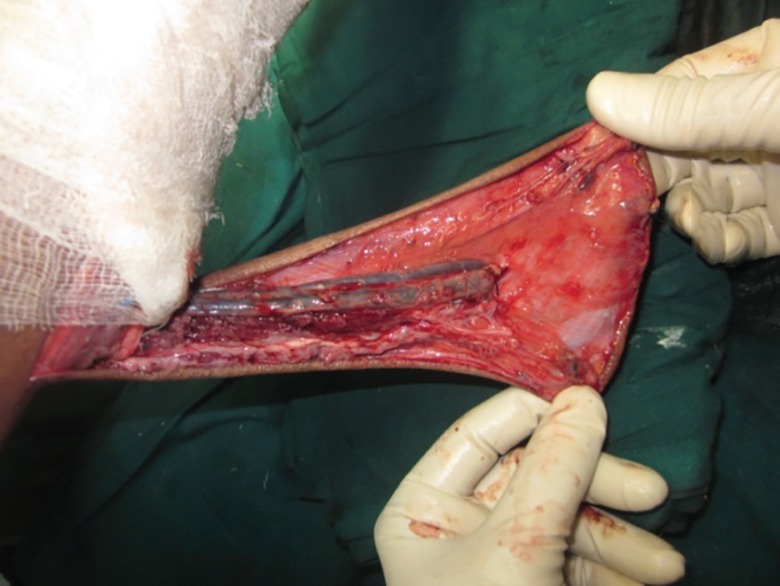
Reverse peroneal artery flap with peroneal vessels in the flap

Branches of the artery going away were coagulated with bipolar or ligated. Sometimes part of flexor hallucis longus muscle was taken with flap if peroneal artery is embedded in muscle (Figure 4 and 5). A good fascial layer is left over tendoachilles. At this stage tourniquet released and flap blood supply checked and complete hemostasis achieved. Flap is then sutured to defect and donor site skin grafted. The pedicle of the flap and raw area of the flap is also skin grafted. An experience of free fibula flap dissection and perforator flap dissection leads to some easiness in dissection of this flap.

**Fig. 6:**
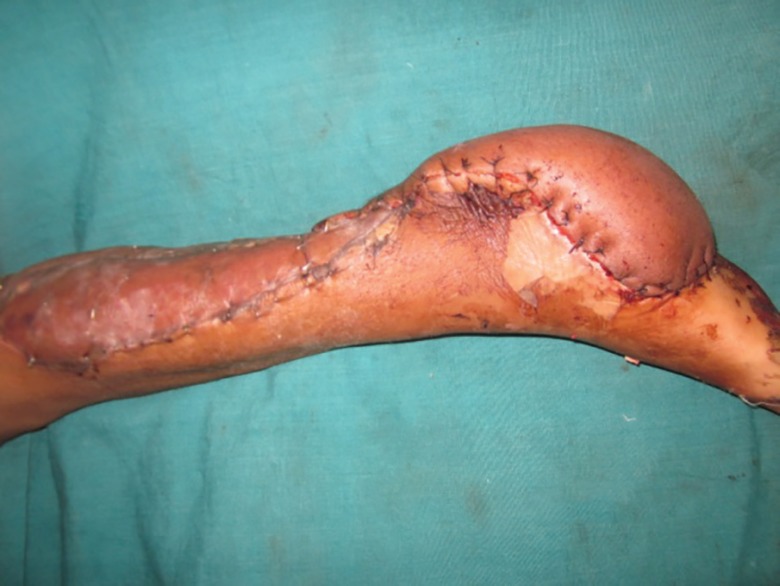
Healthy flap with the almost complete graft uptake seen on 4^th^ postoperative day dressing

**Fig. 7:**
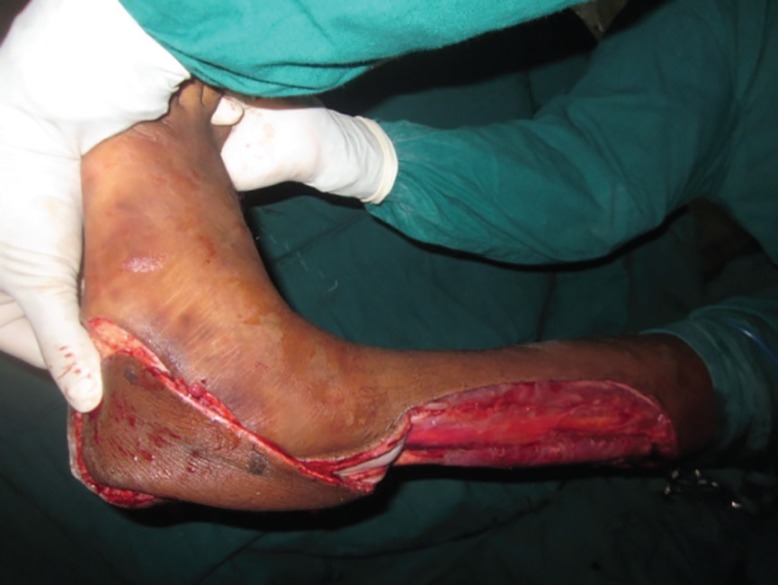
Wide local excision with reverse peroneal artery flap coverage. Peroneal vessels seen in the flap.

**Fig. 8:**
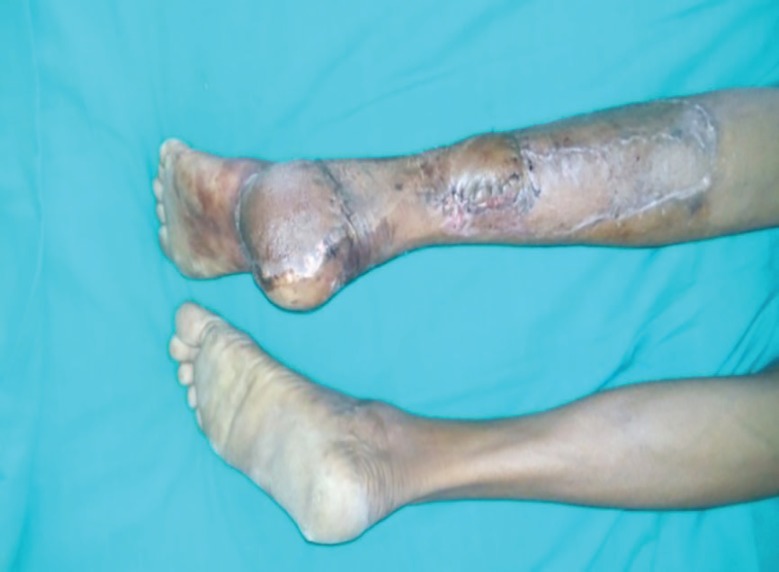
Final appearance after flap inset

We used plaster of Paris slab on dorsal side of leg with some planter flexion at the ankle joint so that flap come in relaxed position. To prevent pedicle compression, extra padding was used over pedicle along with lateral position of the patient on the bed. Two pillows elevation was also given to improve venous outflow and to reduce edema of the flap. In 2 cases we completely islanded the flap and single stage surgery done (Figure 6). In 7 other pedicled flaps second stage of surgery (final inset) done after 18 days of primary surgery under local anesthesia (Figure 7 and 8).In one patient due to deep infection lower 4 cm of fibula get exposed which was removed under spinal anesthesia.

## RESULTS

**Fig. 9:**
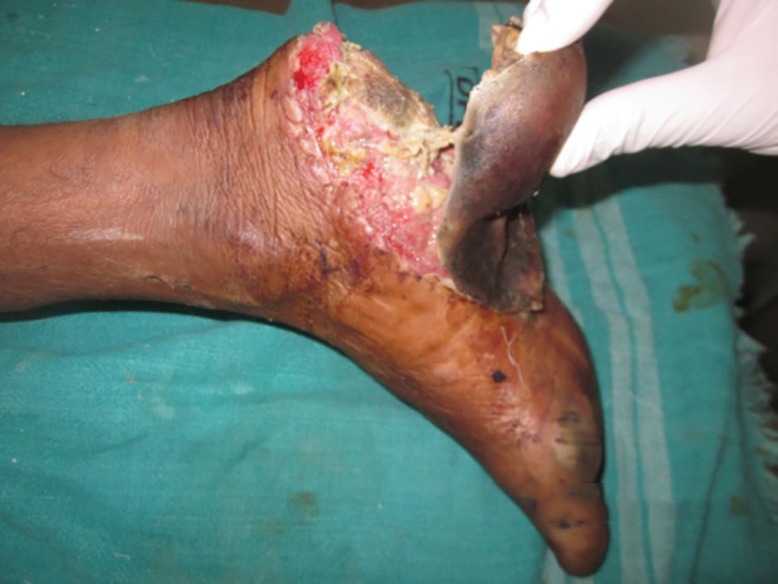
Avulsion of sole up to forefoot due to road traffic accident. Calcaneum bone get exposed here

**Fig. 10:**
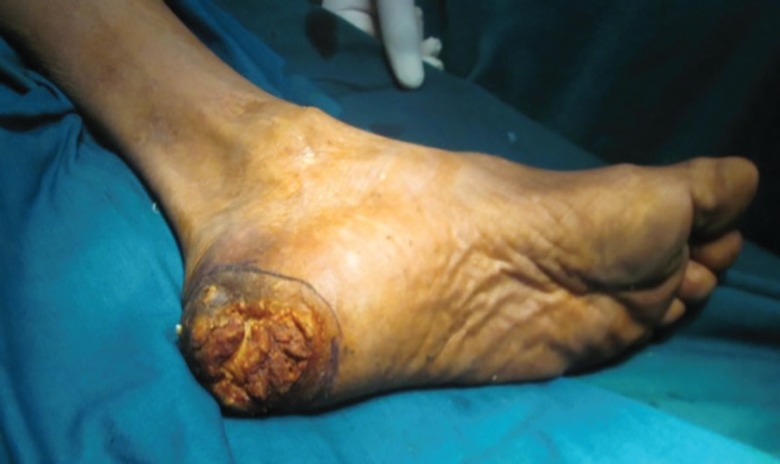
Verrucous carcinoma of the heel underwent wide local excision and reverse peroneal artery flap coverage

**Fig. 11:**
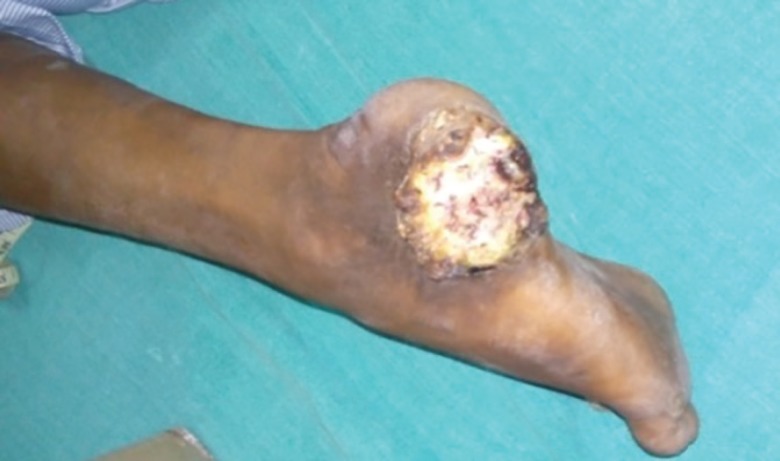
Verrucous carcinoma of the heel region of the foot

Of 10 patients, 6 were men and 4 were women, with an average age of 45 years (35–60 years). Soft tissue defects of variable etiology, like post-traumatic tissue loss (5 cases) (Figure 9), verrucous carcinoma of the foot (3 cases) (Figures 10 and 11) and squamous cell carcinoma (2 cases) were covered. The flap dimensions ranged from 21×10 cm to 28×14 cm in size. All of these flaps were completely survived. There was no incidence of partial flap loss, marginal necrosis or venous congestion in any of these patients.The graft take was generally satisfactory and all the patients were ambulant after 4 weeks following the surgery. 

There were minor problems at the donor site over the lower tendoachilles (graft loss) area in 3 patients, were covered with the reversal of left flap after final inset on postoperative day 18^th ^(Figure 8). In one patient deep infection settled down which leads to 4 cm exposed lower fibular segment. The wound was debrided and 5 cm of bone removed with left flap coverage on 18^th^ day.All donor sites completely healed in 4 weeks. There was no need of regrafting in any patient. After 7-10 days of primary surgery, patient discharged and followed after 18 days for final inset.

**Fig. 12:**
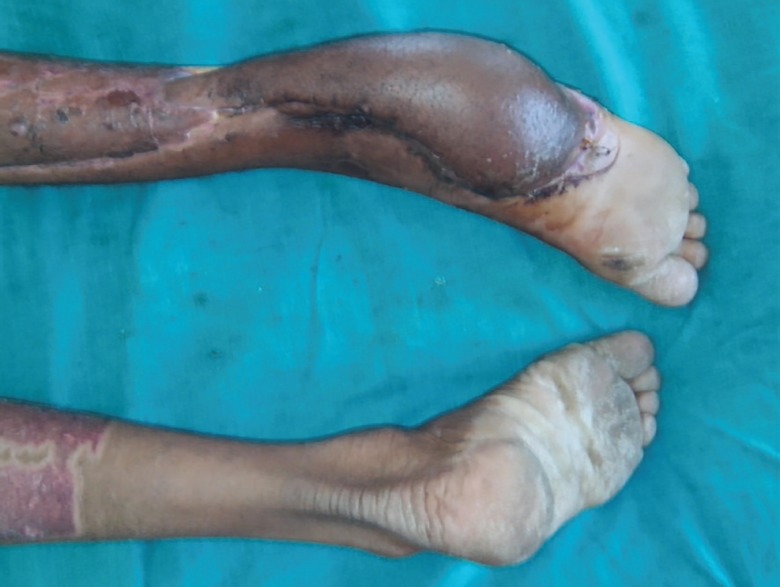
Completely healthy flap on 20 th day of surgery

**Fig. 13:**
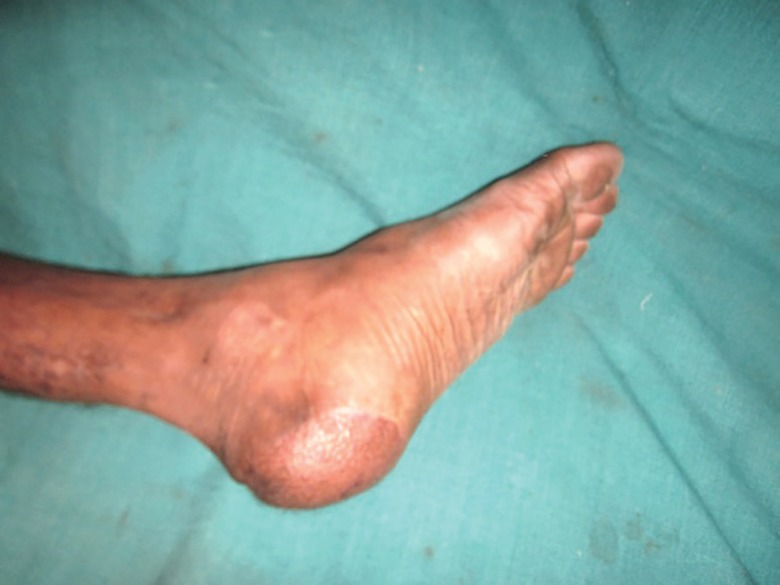
Completely functional and aesthetic look after 3.5 months of the flap surgery

These cases were advised dressings, twice-a-week, on OPD basis. Details of etiology, flap dimensions and complications have been summarized in Table 1. The average period of follow-up was 1 year. Patients were asked to use crepe bandages for 6 months. None of the patients reported problems associated with weight bearing. None of the patient had recurrence of tumor. Aesthetic and functional outcome was good in all patients (Figure 7, 8, 12, and 13). All patients showed their satisfaction for outcome.

**Table 1:** Etiology, defect size, flap dimensions and complications related to the reverse peroneal artery flap

**No.**	**Age/sex**	**Etiology**	**Defect location**	**Flap dimensions (cm)**	**Complications**
1	45/f	Trauma	Heel avulsion	24×11	Partial graft loss at lower donor site
2	45/m	Verrucous carcinoma of heel	Heel	23×10	None
3	42/m	Trauma	Avulsion of heel and sole of midfoot	28×14	Patchy graft loss, healed within 20 days with dressings.
4	38/f	Squamous cell carcinoma of heel	Heel region	26×12	None
5	42/m	Verrucous carcinoma	Heel region	23×10	None
6	60/m	Squamous cell carcinoma of heel	Heel region extending in to the sole	26×11	Partial graft loss at lower donor site
7	43/f	Trauma	Medial malleolus with dorsal foot region	25×10	None
8	48/m	Verrucous carcinoma	Heel region	22×10	Exposed fibula – 5 cm fibula excised and covered with left flap during final inset
9	49/f	Trauma	Medial malleolus region	21×10	None
10	38/m	Trauma	Heel with tendoachillis region	24×11	Partial graft loss at lower donor site

## DISCUSSION

Successful soft tissue reconstruction of large defects over the ankle and foot is often a limb-saving procedure.^4^ Fasciocutaneous or neurofasciocutaneous flaps from the leg are useful and versatile reconstructive options for patients with moderate-sized soft tissue defects of the leg, ankle and foot. Inability to reach the distal defects with limited arc of rotation of wide and short adipofascial pedicles and the precarious venous drainage has been their problems. Although free flap can provide sufficient tissue for reconstruction, not all patients are suitable candidates for free tissue transfer because of the existing comorbidities and economic problems.^8^^-^^10^

This flap design has included reverse flow peroneal artery along with the reverse sural system to achieve more distal and more reliable cover. Authors reporting successful reverse sural flaps are not in thefavour of harvesting flap from the proximal third of the leg.^8^^-^^10^ This neurofasciocutaneous flap is perfused by lower and middle peroneal perforators and has an axial pattern of circulation in the lower 2/3^rd^ of the leg. Flap harvested above this behaves as a random pattern extension of this flap.^8^^-^^10^In our experience with these flaps up to 3/4^th^ of leg can be taken in flap with reliability.

Ayyapan and Chaddha^11^ tried to secure the skin from the proximal third by adding all the connective tissue between the heads of the gastrocnemius (as a “mesentry” containing septocutaneous perforators). This improved the results only marginally, and the study has reported 27% complication rates.Lateral and posterior leg is normally supplied by perforators of peroneal artery reaching the skin via the posterior intermuscular septum. There are constant perforators 7–21 cm from the fibular head.^12^

Perforators were not marked pre-operatively in this study, it may be safer for beginners to do so. In our experience, these perforators were mostly direct septocutaneous perforators. Intramuscular course sometimes present, is through the flexor hallucis longus, and is very short. Thus, dissection of the perforator to the peroneal vessel is quick. Once the perforator has been dissected to the peroneal vessels, the periosteum over the fibula is reflected just proximal to the selected perforator.^13^

This helps in complete visualization of the main pedicle and further dissection. The peroneal vessels are ligated proximally and are reflected distally as necessary for comfortable reach.The presented technique of including the peroneal artery along with its perforator in the (axial system) pedicle acts as arterial supercharging of the flap based on the supramalleolar perforators (routine reverse sural flap) along with increased venus drainage via venae comitantes. This significantly increases the perfusion pressure in the flap taken from the upper third of the leg. When these advantages are used, flap from the upper leg achieves all the advantages of an axial pattern flow.^13^

Harvesting the flap from the upper leg improved the effective pedicle length and helped reaching distal defects up to the bases of the toes. Inclusion of both sural and peroneal systems improves venous drainage and makes the pedicle more substantial to avoid any kink while taking the flap to the foot. We did not encounter any venous or arterial insufficiency in any of the flaps. The maximum flap dimension was 28×14 cm. Irrespective of the large dimensions of the flap, no augmentation procedure like anastomosis of either artery or vein as described by few authors was necessary.^13^

As long as either the posterior or the anterior tibial artery is patent, the use of the peroneal artery does not lead any problems.Reverse radial artery forearm flap is the established method of the reconstruction in cases of injury to wrist and hand, On the contrary, we propose RPAF for reconstruction of injuries to the ankle and foot, preferably where the ankle region is uninjured. Peroneal artery is the least important source of blood supply to the ankle and foot, where the main supply comes from the anterior tibial artery and posterior tibial artery.^14^^,^^15^

The risk to the foot is minimal while successful cover achieved is a major reconstructive achievement in an otherwise difficult area. Even for free tissue transfer in the distal extremities, most of the surgeons prefer end-to-end anastomosis (privileged communication with multiple authors), ultimately losing one of the major vessels supplying to ankle and foot region. We did not observe any ischemia-related complications in the leg attributable to harvest of the RPAF. The increased amount of soft tissue transferred, the more distal reach, increased reliability and ease of rotation compared with any other regional flap including the sural flap helped us in the salvage of these limbs without need of microvascular surgery.^14^

Small disadvantage of secondary procedure of final inset is there, but due to this tendoachilis region and foot are more aesthetic in appearance rather than in single sitting procedure. and Peroneal artery is least likely to have atherosclerosis.^14^This flap offers the following multiple advantages over other distally based fasciocutaneous flaps including (i) reliable flap dimensions can be extended up to the 3/4^th^ of leg (ii) pedicle length can be planned liberally to avoid kinking, and (iii) with longer pedicle, the flap can reach more distal areas over the sole.^14^

This modified flap can be suitable for (i) soft tissue defects of large dimensions, especially over the heel, distal sole and the dorsum of the foot, (ii) patients with more distal defects where the standard reverse sural flaps may not reach, (iii) patients with large raw area around the ankle and foot, where the routine reverse flap may not be adequate and (iv) salvage in case of failed free flap. All of the procedures were carried out under spinal anesthesia, and the average operating time was around 2.5 hours.^14^

The initial 4 cases done under supine position but after that we realize and found that prone position is far better for defect preparation, flap dissection, flap insetting and donor site grafting. Post-operative positioning was simple and patients tolerated the position well. Second surgery was done after 18 days of the primary surgery under local anesthesia. In all of the presented cases, this flap has helped in avoiding microvascular surgery.^14^

In our experience, extension of reverse sural flaps up to 3/4^th^of the leg as RPAF was safe and reliable. These promising results help us to refine our techniques to deliver reliable cover in the distal foot defects. Upper extension of the flap adds versatility in planning and tensionless reach of the flap to the recipient defect. In most of these cases, free flap was the only other option. Under these circumstances, RPAF has been used with very good results for reconstruction of heel and distal sole defects of larger dimensions.

## CONFLICT OF INTEREST

The authors declare no conflict of interest.
